# Effect of human bone marrow mesenchymal stromal cells on cytokine production by peripheral blood naive, memory, and effector T cells

**DOI:** 10.1186/scrt537

**Published:** 2015-01-05

**Authors:** Paula Laranjeira, Monia Pedrosa, Susana Pedreiro, Joana Gomes, Antonio Martinho, Brigida Antunes, Tania Ribeiro, Francisco Santos, Helder Trindade, Artur Paiva

**Affiliations:** Blood and Transplantation Center of Coimbra, Portuguese Institute of the Blood and Transplantation, Quinta da Vinha Moura, São Martinho do Bispo, 3041-861 Coimbra, Portugal; Signal Transduction Laboratory, Center of Cellular Biology, SACS and Departament of Biology, University of Aveiro, Campus Universitário de Santiago, 3810-193 Aveiro, Portugal; QOPNA, Department of Chemistry, University of Aveiro, Campus Universitário de Santiago, 3810-193 Aveiro, Portugal; Cell2B Advanced Therapeutics SA, Biocant Park, Núcleo 04, Lote 4A, 3060-197 Cantanhede, Portugal

## Abstract

**Introduction:**

The different distribution of T cells among activation/differentiation stages in immune disorders may condition the outcome of mesenchymal stromal cell (MSC)-based therapies. Indeed, the effect of MSCs in the different functional compartments of T cells is not completely elucidated.

**Methods:**

We investigated the effect of human bone marrow MSCs on naturally occurring peripheral blood functional compartments of CD4^+^ and CD8^+^ T cells: naive, central memory, effector memory, and effector compartments. For that, mononuclear cells (MNCs) stimulated with phorbol myristate acetate (PMA) plus ionomycin were cultured in the absence/presence of MSCs. The percentage of cells expressing tumor necrosis factor-alpha (TNF-α), interferon gamma (IFNγ), and interleukin-2 (IL-2), IL-17, IL-9, and IL-6 and the amount of cytokine produced were assessed by flow cytometry. mRNA levels of IL-4, IL-10, transforming growth factor-beta (TGF-β), and cytotoxic T-lymphocyte-associated protein 4 (CTLA4) in purified CD4^+^ and CD8^+^ T cells, and phenotypic and mRNA expression changes induced by PMA + ionomycin stimulation in MSCs, were also evaluated.

**Results:**

MSCs induced the reduction of the percentage of CD4^+^ and CD8^+^ T cells producing TNF-α, IFNγ, and IL-2 in all functional compartments, except for naive IFNγ^+^CD4^+^ T cells. This inhibitory effect differentially affected CD4^+^ and CD8^+^ T cells as well as the T-cell functional compartments; remarkably, different cytokines showed distinct patterns of inhibition regarding both the percentage of producing cells and the amount of cytokine produced. Likewise, the percentages of IL-17^+^, IL-17^+^TNF-α^+^, and IL-9^+^ within CD4^+^ and CD8^+^ T cells and of IL-6^+^CD4^+^ T cells were decreased in MNC-MSC co-cultures. MSCs decreased IL-10 and increased IL-4 mRNA expression in stimulated CD4^+^ and CD8^+^ T cells, whereas TGF-β was reduced in CD8^+^ and augmented in CD4^+^ T cells, with no changes for CTLA4. Finally, PMA + ionomycin stimulation did not induce significant alterations on MSCs phenotype but did increase indoleamine-2,3-dioxygenase (IDO), inducible costimulatory ligand (ICOSL), IL-1β, IL-8, and TNF-α mRNA expression.

**Conclusions:**

Overall, our study showed that MSCs differentially regulate the functional compartments of CD4^+^ and CD8^+^ T cells, which may differentially impact their therapeutic effect in immune disorders. Furthermore, the influence of MSCs on IL-9 expression can open new possibilities for MSC-based therapy in allergic diseases.

**Electronic supplementary material:**

The online version of this article (doi:10.1186/scrt537) contains supplementary material, which is available to authorized users.

## Introduction

The discovery of the immunosuppressive potential of mesenchymal stromal cells (MSCs) propelled a large number of studies in the past decade, mainly focusing on T cells. The suppressive effect of MSCs over T cells comprises inhibition of T-cell proliferation, activation, differentiation in effector cells, and effector function by altering their cytokine profile and impairing the cytolytic activity of cytotoxic T cells [1]. MSC-derived immunosuppression can be achieved by direct MSC-T cell interaction, through plasmatic membrane proteins or soluble factors produced by MSCs, or indirectly by MSC-mediated suppression of antigen-presenting cells [2]. In fact, human bone marrow (BM) MSCs impair dendritic cell maturation and decrease the expression of co-stimulatory molecules and interleukin-12 (IL-12) while increasing IL-10 expression and consequently hampering T-cell activation [2–6]. A similar effect is observed in monocytes which, in the presence of human BM-MSCs, develop an anti-inflammatory phenotype with increased IL-10 expression [7–9].

However, it is well established that the behavior of MSCs depends on numerous factors, such as the source of MSCs, the type of immune cells present in the cell culture, the state of activation and differentiation of the T cells, and the type of stimuli used [10–14]. In turn, the information available on the effect of MSCs over T cells at different stages of activation/differentiation is scarce, and the data concerning the influence of MSCs on the naive-effector T-cell differentiation process are contradictory. Most of the publications describe an inhibitory action over Th1 and Th17 differentiation, along with a decreased expression of the cytokines related to these effector phenotypes—interferon gamma (IFNγ), IL-2, and tumor necrosis factor-alpha (TNF-α) for Th1; and IL-17A, IL-17F, and IL-21 for Th17—both *in vitro* and *in vivo*[1, 14–19]. Nevertheless, some studies reported that MSCs promote Th17 differentiation and IL-17A production [1, 20–23].

Different disorders of the immune system and different stages of each immune disease are characterized by distinctive distribution of T cells among activation/differentiation compartments, which is a reflection of the T-cell subsets with a predominant role in the disease etiology. This highlights the importance of knowing the effect of MSCs on each individual functional compartment in order to predict and understand the outcome of MSC cell therapy.

Here, we investigate the suppressive effect of human BM-MSCs over peripheral blood CD4^+^ and CD8^+^ T cells distributed among the naturally occurring naive, central memory (CM), effector memory (EM), and effector compartments. Peripheral blood mononuclear cells (MNCs) were stimulated with phorbol myristate acetate (PMA) plus ionomycin in the absence/presence of MSCs, and the expression of IL-2, TNF-α, and IFNγ was evaluated by flow cytometry for CD4^+^ and CD8^+^ T cells distributed among the naive, CM, EM, and effector compartments; the expression of IL-6, IL-9, and IL-17 was also evaluated in total CD4^+^ and CD8^+^ T cells. In addition, IL-4, IL-10, transforming growth factor-beta (TGF-β), and T-lymphocyte-associated protein 4 (CTLA4) mRNA levels were assessed in fluorescence-activated cell sorting (FACS)-purified CD4^+^ and CD8^+^ T cells. Finally, the protein expression of CD13, CD44, CD73, CD90, CD106, and CD184 (CXCR4) and mRNA transcripts of indoleamine-2,3-dioxygenase (IDO), inducible costimulatory ligand (ICOSL), TGF-β1, IL-1β, IL-8, and TNF-α were evaluated in non-stimulated MSCs and after stimulation with PMA plus ionomycin.

In this study, we report that the functional compartments of CD4^+^ and CD8^+^ T cells are differentially regulated by MSCs, which may condition the outcome of MSC cell therapy in immune disorders with distinctive distribution of T cells among activation/differentiation compartments. Of note, MSCs decrease the percentage of CD4^+^ and CD8^+^ T cells expressing TNF-α, IFNγ, IL-2, IL-6, IL-9, and IL-17 as well as the amount of TNF-α and IFNγ produced at a single-cell level. The influence of MSCs on IL-9 can be a possible mechanism contributing to allergic inflammation amelioration by MSCs.

## Materials and methods

### Collection and isolation of peripheral blood mononuclear cells and bone marrow mesenchymal stromal cells

Peripheral blood samples from a total of eight healthy donors (one male and seven females; mean age of 41 ± 11 years, ranging from 21 to 50 years old), collected in heparin at the Blood and Transplantation Center of Coimbra (Portugal), and human BM samples from healthy donors (age ranging from 20 to 40 years old) were included in the present study. The use of these biological samples for research purpose was approved by the ethics committee of Instituto Português de Oncologia de Lisboa Francisco Gentil (laws 97/95 and 46/2004), and all participants gave written informed consent before entering in the study.

Peripheral blood MNCs were isolated by Lymphoprep (Stemcell Technologies, Vancouver, BC, Canada) gradient density centrifugation and then washed twice in Hank’s Balanced Salt Solution (Gibco, Life Technologies, Paisley, UK). The MNCs pellet was resuspended in RPMI 1640 with GlutaMax medium (Invitrogen, Life Technologies) with antibiotic-antimycotic (Gibco) to the final concentration of 10^6^ cells/mL.

Peripheral blood MNCs were subsequently analyzed for protein and mRNA expression in the following experimental conditions: (1) 0.5 × 10^6^ MNCs + 500 μL RPMI (negative control), (2) 0.5 × 10^6^ MNCs + 0.25 × 10^6^ MSCs (negative control), (3) 0.5 × 10^6^ MNCs + 500 μL RPMI + PMA + ionomycin (positive control), and (4) 0.5 × 10^6^ MNCs + 0.25 × 10^6^ MSCs + PMA + ionomycin. All of the aforementioned cell cultures were carried out for 20 hours at 37°C, in a sterile environment with 5% CO_2_ and humidified atmosphere, plus an incubation period of 4 hours with the stimulator agents. The cell culture and stimulation protocols are detailed below in the “Immunophenotypic study of mesenchymal stromal cells and peripheral blood T cells” section.

For the isolation of human BM-MSCs, MNCs were isolated from BM samples by using a Sepax S-100 system (Biosafe, Eysins, Switzerland) in accordance with the instructions of the manufacturer. Cell number and viability were determined using the Trypan Blue (Gibco) exclusion method.

BM MNCs were plated at a density of 2 × 10^5^ cells/cm^2^ in Dulbecco’s modified Eagle’s medium (Gibco) supplemented with 10% qualified fetal bovine serum (Sigma-Aldrich). After a 3-day incubation at 37°C in humidified atmosphere containing 5% CO_2_, the non-adherent cell fraction was discarded, and the adherent culture was maintained with a complete medium renewal every 3 to 4 days. After reaching a 70% to 80% confluency cells were detached by using TrypLE (Life Technologies) for 7 minutes and then replated at an initial density of 3,000 cells/cm^2^. For this study, MSCs passages 3 and 5 were used.

MSCs identity was confirmed by performing fluorescent morphological analysis, mesodermal differentiation assays (osteogenic, adipogenic, and chondrogenic), and immunophenotype characterization as described by Dominici *et al*. [24]. Subsequently, MSCs were resuspended in RPMI 1640 with GlutaMax medium (Invitrogen) with antibiotic-antimycotic (Gibco) to the final concentration of 0.5 × 10^6^ cells/mL. The protein and mRNA expression of MSCs was studied in the following experimental conditions: (1) 0.25 × 10^6^ MSCs + 500 μL RPMI (non-stimulated MSCs) and (2) 0.25 × 10^6^ MSCs + 500 μL RPMI + PMA + ionomycin. All of the aforementioned cell cultures were carried out for 20 hours at 37°C, in a sterile environment with 5% CO_2_ and humidified atmosphere, plus an incubation period of 4 hours with the stimulator agents. The cell culture and stimulation protocols are detailed below, in “Immunophenotypic study of mesenchymal stromal cells and peripheral blood T cells” section.

### Immunophenotypic study of mesenchymal stromal cells and peripheral blood T cells

#### Mesenchymal stromal cell stimulation with phorbol myristate acetate plus ionomycin

For the immunophenotypic study of MSCs, we plated in four wells of tissue culture plates (Falcon, Becton Dickinson Biosciences, BD, San Jose, CA, USA) 0.25 × 10^6^ MSCs in 1 mL of RPMI 1640 with GlutaMax medium (Invitrogen) with antibiotic-antimycotic (Gibco). MSCs were cultured for 20 hours at 37°C in a sterile environment with 5% CO_2_ and humidified atmosphere (to be in the same experimental conditions than as those MSCs co-cultured with MNCs). MSCs from two of the four wells were stimulated with PMA (50 ng/mL; Sigma-Aldrich) plus ionomycin (1 μg/mL; Boehringer Mannheim, Germany) for 4 hours at 37°C in humidified atmosphere containing 5% CO_2_; in the remaining two wells, MSCs were not stimulated. The immunophenotypic study and mRNA expression quantification were performed in non-stimulated and PMA + ionomycin-stimulated MSCs.

#### Immunophenotypic study of mesenchymal stromal cells

For each experimental condition tested, cells were detached by using TrypLE (Gibco); after incubating for 10 minutes at −20°C, the content of each well was transferred to a 12 × 75 mm polystyrene cytometer tube, centrifuged for 5 minutes at 540 *g* and the supernatant was discarded. MSCs immunophenotype was assessed by using the seven-color monoclonal antibody (mAb) combination detailed in Table 1, tube 1. The cell pellet was incubated with the mAb for 10 minutes in the darkness and washed with phosphate-buffered saline (PBS). Finally, cells were resuspended in 500 μL of PBS and immediately acquired in a FACSCanto II (BD) flow cytometer.

**Table 1:** **Panel of monoclonal antibody reagents (with clones and commercial source) used for the immunophenotypic characterization of mesenchymal stromal cell**s **and peripheral blood T cells**

Fluorochrome								
Tube	PacB	PacO	FITC	PE	PerCPCy5.5 or PECy5	PECy7	APC	APCH7
**1**	**CD44**	**CD45**	**CD106**	**CD73**	**CD184**	**CD13**	**CD90**	
	Clone IM7 Biolegend	HI30 Invitrogen	51-10C9 BD Pharmingen	AD2 BD Pharmingen	12G5 BD	Immu103.44 Beckman Coulter	5E10 BD Pharmingen	
**2**	**CD3**		**cyTNF-α**	**cyIL-17**	**CD27**	**CD4**	**CD45RA**	**CD8**
	UCHT1 BD Pharmingen		MP6-XT22 BD Pharmingen	SCPL1362 BD Pharmingen	14CD27 Beckman Coulter	SFCI12T4D11 Beckman Coulter	HI100 BD	SK1 BD
**3**	**CD3**		**cyIFNγ**	**cyIL-6**	**CD27**	**CD4**	**CD45RA**	**CD8**
	UCHT1 BD Pharmingen		4S.B3 BD Pharmingen	MQ2-6A3 BD Pharmingen	14CD27 Beckman Coulter	SFCI12T4D11 Beckman Coulter	HI100 BD	SK1 BD
**4**	**CD3**		**cyIL-2**	**cyIL-9**	**CD27**	**CD4**	**CD45RA**	**CD8**
	UCHT1 BD Pharmingen		MQ1-17H12 BD	MH9A3 BD Pharmingen	14CD27 Beckman Coulter	SFCI12T4D11 Beckman Coulter	HI100 BD	SK1 BD
**5**	**CD4**		**CD25**	**CD127**		**TCRγδ**	**CD8**	**CD3**
	RPA-T4 BD Pharmingen		M-A251 BD Pharmingen	R34.34 Beckman Coulter		11 F2 BD Pharmingen	B9.11 Beckman Coulter	SK7 BD Pharmingen

Commercial sources: Biolegend (San Diego, CA, USA), Invitrogen, Life Technologies (Carlsbad, CA, USA), BD Pharmingen (San Diego, CA, USA), BD (Becton Dickinson Biosciences, San Jose, CA, USA), Beckman Coulter (Miami, FL, USA). APC, allophycocyanin; APCH7, allophycocyanin-hilite 7; FITC, fluorescein isothiocyanate; mAb, monoclonal antibody; MSC, mesenchymal stromal cell; PacB, pacific blue; PacO, pacific orange; PE, phycoerythrin; PECy5, phycoerythrin-cyanine 5; PECy7, phycoerythrin-cyanine 7; PerCPCy5.5, peridinin chlorophyll protein-cyanine 5.5.

#### Co-culture of peripheral blood mononuclear cells and mesenchymal stromal cells and in vitro stimulation with phorbol myristate acetate plus ionomycin

In four wells of tissue culture plates (Falcon), 0.5 × 10^6^ MNCs were plated in 1 mL of RPMI 1640 with GlutaMax medium (Invitrogen) with antibiotic-antimycotic (Gibco), and in four other wells of tissue culture plates (Falcon), we plated 0.5 × 10^6^ MNCs + 0.25 × 10^6^ MSCs in a final volume of 1 mL, establishing a ratio of 2:1 (MNCs:MSCs). Cells were cultured for 20 hours at 37°C in a sterile environment with 5% CO_2_ and humidified atmosphere.

After the incubation period, PMA + ionomycin (50 ng/mL and 1 μg/mL, respectively) was added to two wells with MNCs and two wells with co-cultured MNCs + MSCs; the cells in the remaining wells (two with MNCs and two with MNCs + MSCs) were not stimulated. Brefeldin A (10 μg/mL) from *Penicillium brefeldiamun* (Sigma-Aldrich) was added to one well of each experimental condition—(1) MNCs, (2) MNCs + MSCs, (3) MNCs + PMA + ionomycin, and (4) MNCs + MSCs + PMA + ionomycin—to prevent the release of *de novo* produced cytokines outside the cells. We proceeded to an incubation at 37°C in a sterile environment with 5% CO_2_ humidified atmosphere for 4 hours.

The samples with brefeldin A were used for the study of cytokine expression in T cells by flow cytometry, while the mRNA expression was performed in the samples without brefeldin A. All of the aforementioned protein and mRNA expression studies were performed in all of the different culture conditions: MNCs, MNCs + MSCs, MNCs + PMA + ionomycin, and MNCs + MSCs + PMA + ionomycin.

#### Immunophenotypic study of peripheral blood T cells

For each experimental condition tested, cells were detached by using TrypLE (Gibco); after incubating for 10 minutes at −20°C, the content of each well was transferred to a 12 × 75 mm polystyrene cytometer tube, centrifugedv for 5 minutes at 540 *g* and the supernatant was discarded. Immunophenotypic analysis of peripheral blood T cells, cultured in the presence/absence of PMA + ionomycin and in the presence/absence of MSCs, was performed by using seven-color mAb combinations, detailed in Table 1. In short, cells were stained with the mAb for surface proteins antigens (CD3, CD27, CD4, CD45RA, and CD8) and, after an incubation period of 10 minutes in the dark at room temperature, washed with PBS. For intracellular staining, Fix&Perm (Caltag, Hamburg, Germany) reagent was used, in accordance with the instructions of the manufacturer and in parallel with the mAb for TNF-α and IL-17 (tube 2), IFNγ and IL-6 (tube 3), or IL-2 and IL-9 (tube 4). After washing twice with PBS, the cell pellet was resuspended in 500 μL of PBS and immediately acquired.

#### Data acquisition and analysis

Data acquisition were performed in a FACSCanto™II (BD) flow cytometer equipped with FACSDiva software (version 6.1.2; BD). For both MSCs and MNCs immunophenotypic studies, the whole sample from each tube was acquired and stored, corresponding to a number of events always above 0.1 × 10^6^ or 0.5 × 10^6^ events, respectively. For data analysis, Infinicyt (version 1.7) software (Cytognos SL, Salamanca, Spain) was used.

#### Immunophenotypic identification of T cells within the different functional compartments

The identification and quantification of the four T-cell functional compartments were carried out after the exclusion of cell debris (which corresponded to events with very low forward scatter (FSC) and heterogeneous side scatter (SSC) light dispersion properties) and doublets (identified by their FSC area and FSC height characteristics). T cells were identified on the basis of CD3 positivity and intermediate FSC and SSC properties (Additional file 1: Figure S1). Within this cell population, the functional compartments of CD4^+^ and CD8^+^ T cells (phenotypically characterized as CD3^+^CD4^+^CD8^−^ and CD3^+^CD4^−^CD8^+^, respectively) were identified according to their differential expression of CD45RA and CD27 as follows: naive T cells are characterized by CD45RA^+^CD27^+^ expression, CM T cells are CD45RA^−^CD27^+^, and EM and effector T cells display CD45RA^−^CD27^−^ and CD45RA^+^CD27^−^ immunophenotype, respectively (Additional file 1: Figure S1).

### Cell purification by fluorescence-activated cell sorting

CD4^+^ and CD8^+^ T-cell populations from the cell cultures were purified by FACS (using a FACSAria II flow cytometer; BD) according to their typical phenotype. Thus, the six-color mAb combination used (Table 1, tube 5) allowed the identification of CD4^+^ T cells (CD3^+^CD4^+^CD8^−^TCRγδ^−^) and CD8^+^ T cells (CD3^+^CD4^−^CD8^+^TCRγδ^−^). The remaining mAbs found in the panel were used to exclude γδ T cells (which correspond to CD3^+^TCRγδ^+^ events) and regulatory T (Treg) cells (which are CD25^++^CD127^−/dim^). The purified cell populations were subsequently used for the quantification of mRNA expression.

### Analysis of mRNA expression in mesenchymal stromal cells and purified CD4^+^ and CD8^+^ T cells

The content of each well of cultured MSCs under the different experimental condition tested, or the purified CD4^+^ and CD8^+^ T-cell populations, were transferred to a 1.5-mL Eppendorf tube and centrifuged for 5 minutes at 300 *g*, and the pellet was resuspended in 350 μL of RLT Lysis Buffer (Qiagen, Hilden, Germany). Total RNA was extracted with the RNeasy Micro kit (Qiagen) in accordance with the instructions of the supplier. Total RNA was eluted in a 20-μL volume of RNase-free water. RNA was reverse-transcribed with Tetra cDNA Synthesis (Bioline, London, UK) in accordance with the instructions of the manufacturer. Relative quantification of gene expression by real-time polymerase chain reaction (PCR) was performed in the LightCycler 480 II (Roche Diagnostics, Rotkreuz, Switzerland). Real-time PCRs were carried out by using 1x QuantiTect SYBR Green PCR Master Mix (Qiagen) and 1x QuantiTect Primer Assay (for MSCs: TNF3: QT01079561; IL-8: QT00000322; IL-1β: QT00021385; TGF-β1: QT00025718; ICOSL: QT00023660; IDO: QT00000504; for purified CD4+ and CD8+ T cells: IL-10: QT00041685; IL-2: QT00015435; IL-4: QT00012565; TGF-β: QT00000728; CTLA4: QT01670550) (Qiagen) in a final volume of 10 μL. The reactions were performed by using the following thermal profile: one cycle of 10 minutes at 95°C, 50 cycles of 10 seconds at 95°C, 20 seconds at 55°C and 30 seconds at 72°C, one cycle of 5 seconds at 95°C, 1 minute at 65°C and continuo at 97°C, and one cycle of 10 seconds at 21°C. All samples were run in duplicate. Real-time PCR results were analyzed with the LightCycler software (Roche Diagnostics). GeNorm software (PrimerDesign Ltd., Southampton, UK) was used to select the reference genes to normalize data. The reference genes used for gene expression analysis were cytochrome c1 (CYC1) and splicing factor 3a subunit 1 (SF3A1) for MSCs and beta-2 microglobulin (B2M) and tyrosine 3-monooxygenase/tryptophan 5-monooxygenase activation protein, zeta (YWHAZ) for CD4^+^ and CD8^+^ T cells. The normalized expression levels of the genes of interest were calculated by using the delta Ct (change in threshold cycle) method.

### Statistical analysis

To determine the statistic significance of the differences observed between different culture conditions, non-parametric Wilcoxon and Friedman’s paired-sample tests were performed by using Statistical Package for Social Sciences (IBM SPSS, version 17.0; IBM Corporation, Armonk, NY, USA). Data were expressed as mean percentage ± standard deviation. Statistically significant differences were considered when the *P* value was lower than 0.05.

## Results

To investigate the ability of MSCs to regulate cytokine protein expression in CD4^+^ and CD8^+^ T cells and how the influence of MSCs varies among the different T-cell functional compartments (naive, CM, EM, and effector compartment), non-stimulated or PMA + ionomycin-stimulated MNCs were cultured in the absence or presence of MSCs. We used eight-color flow cytometry to identify the four abovementioned compartments among CD4^+^ and CD8^+^ T cells and analyze the protein expression of TNF-α, IFNγ, and IL-2 within each cell compartment and of IL-17, IL-6, and IL-9 in total CD4^+^ or CD8^+^ T cells or both. Besides, mRNA expression of IL-4, IL-10, TGF-β, and CTLA4 in purified CD4^+^ and CD8^+^ T cells was also assessed. Finally, MSCs were stimulated with PMA plus ionomycin, and the protein expression of CD13, CD44, CD73, CD90, CD106, and CD184 (CXCR4) and mRNA levels of IDO, ICOSL, TGF-β, IL-1β, IL-8, and TNF-α were evaluated and compared with that of non-stimulated MSCs.

### Mesenchymal stromal cells decrease the frequency of T cells producing TNF-α, IFNγ, and IL-2 as well as the protein expression at single-cell level and regulate differentially the distinct T-cell functional compartments

**Figure 1:**
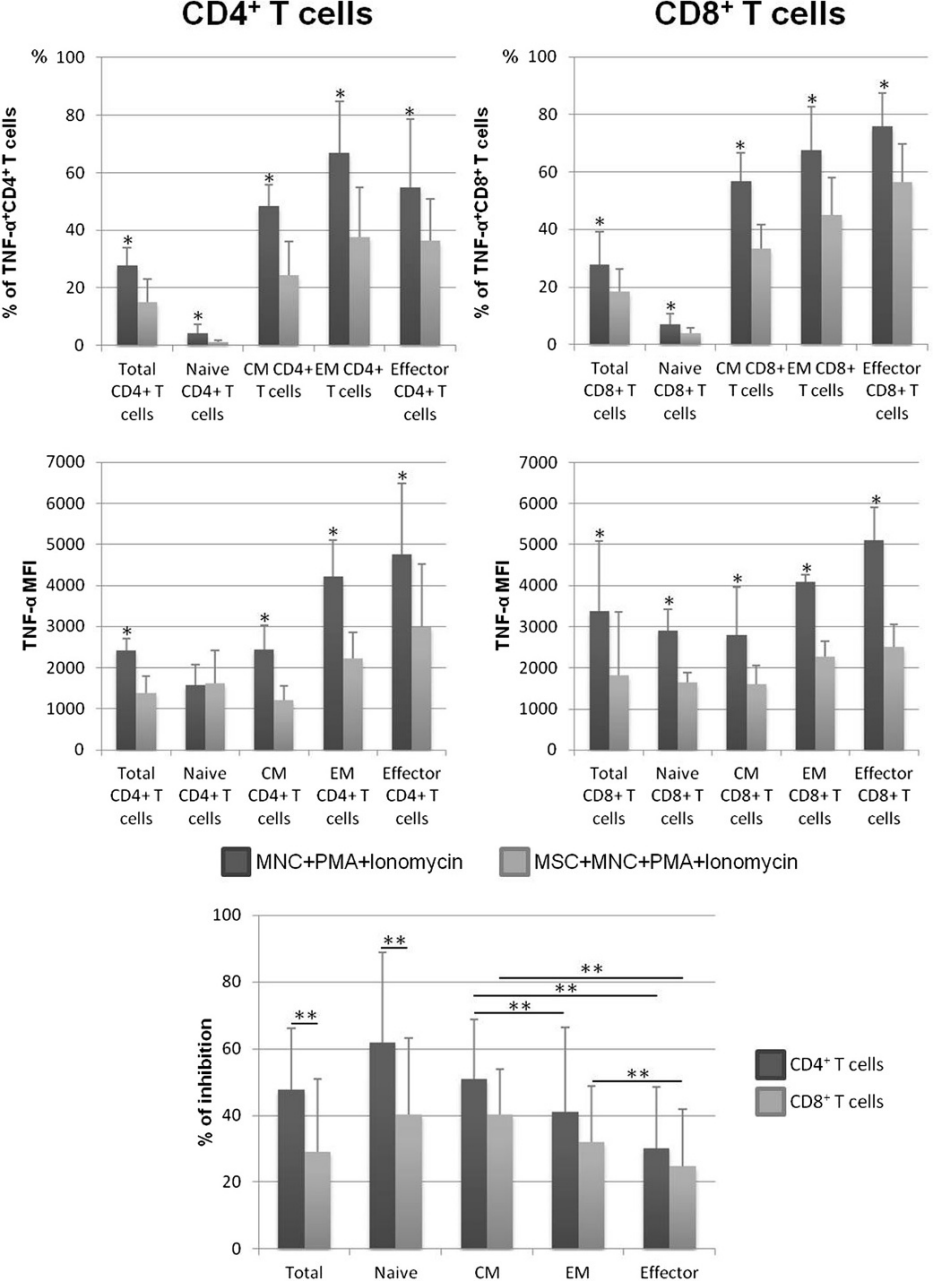
**Percentage of CD4** ^**+**^ **and CD8** ^**+**^ **T cells expressing tumor necrosis factor-alpha (TNF-α).** The upper panels show the percentage (mean ± standard deviation) of CD4^+^ and CD8^+^ T cells, distributed among their functional compartments, producing TNF-α (within the correspondent functional compartment of CD4^+^ or CD8^+^ T cells, respectively) and amount of protein expressed per cell, measured as mean fluorescence intensity (MFI) (mean ± standard deviation), after mononuclear cell (MNC) stimulation with phorbol myristate acetate (PMA) plus ionomycin in the absence of mesenchymal stromal cells (MSCs) (MNCs + PMA + ionomycin) or in co-culture with MSCs (MSCs + MNCs + PMA + ionomycin). The lower panel represents the percentage of inhibition induced by MSCs on the percentage of CD4^+^ and CD8^+^ T cells expressing TNF-α. Statistically significant differences were considered when *P* <0.05 for Wilcoxon paired-sample test: *versus MSCs + MNCs + PMA + ionomycin; **P <0.05 for the groups indicated in the figure. CM, central memory; EM, effector memory.

Co-culture of MSCs with PMA + ionomycin-stimulated MNCs decreased the percentage of both CD4^+^ and CD8^+^ T cells expressing TNF-α (*P* <0.05)—observed in all individuals enrolled in this study—as well as the amount of protein produced per cell (*P* <0.05), measured as mean fluorescence intensity (MFI), in all cell compartments analyzed, except for TNF-α MFI in naive CD4^+^ T cells, where MSCs did not induce changes (Figure 1). Of note, the effect of MSCs over the frequency of TNF-α^+^ cells was more pronounced in CD4^+^ T cells, compared with CD8^+^ T cells, and in naive and CM compartments for both CD4^+^ and CD8^+^ T cells (Figure 1).

**Figure 2:**
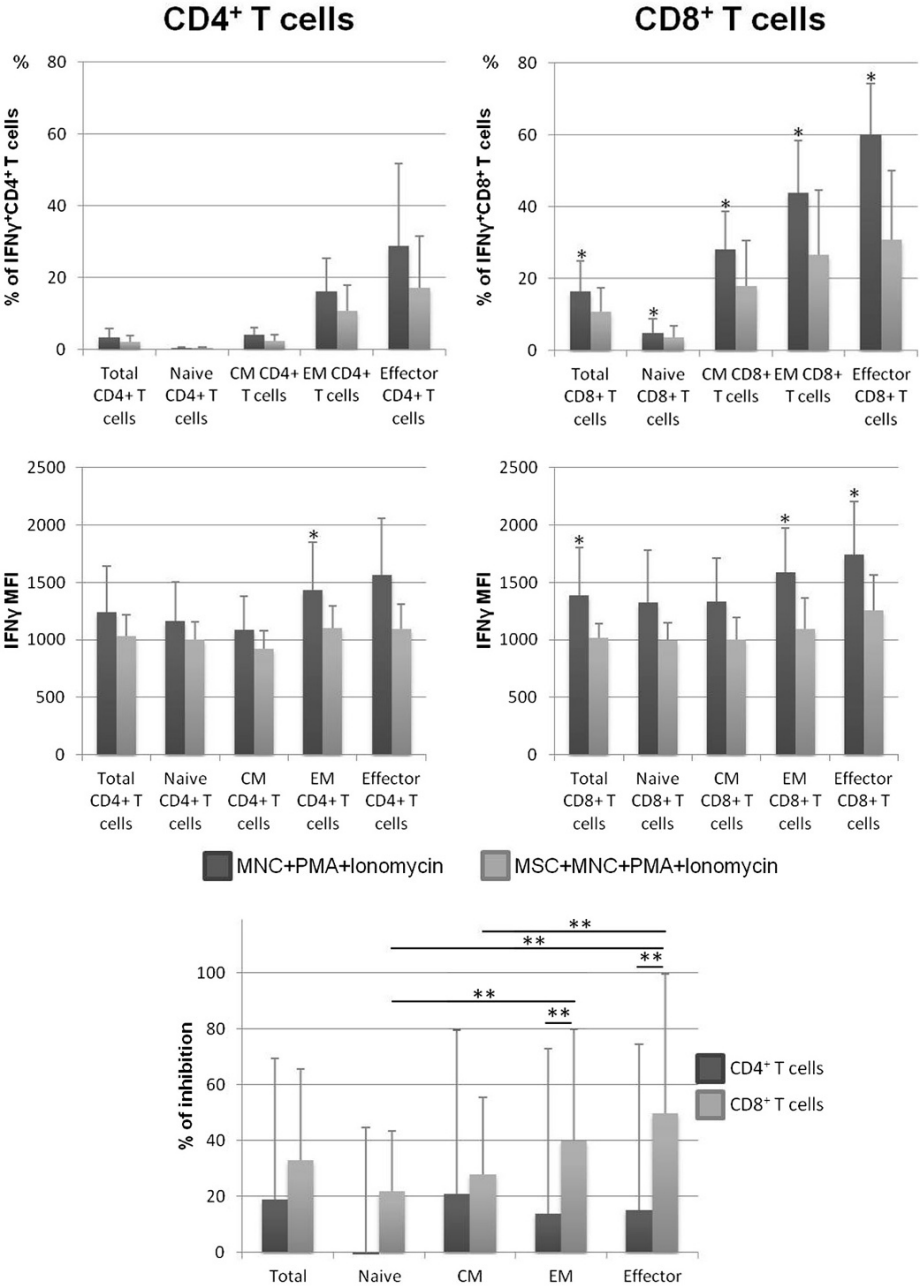
**Percentage of CD4** ^**+**^ **and CD8** ^**+**^ **T cells expressing interferon gamma (IFNγ).** The upper panels show the percentage (mean ± standard deviation) of CD4^+^ and CD8^+^ T cells, distributed among their functional compartments, producing IFNγ (within the correspondent functional compartment of CD4^+^ or CD8^+^ T cells, respectively) and amount of protein expressed per cell, measured as mean fluorescence intensity (MFI) (mean ± standard deviation), after mononuclear cell (MNC) stimulation with phorbol myristate acetate (PMA) plus ionomycin in the absence of mesenchymal stromal cells (MSCs) (MNCs + PMA + ionomycin) or in co-culture with MSCs (MSCs + MNCs + PMA + ionomycin). The lower panel represents the percentage of inhibition induced by MSCs on the percentage of CD4^+^ and CD8^+^ T cells expressing IFNγ. Statistically significant differences were considered when *P* <0.05 for Wilcoxon paired-sample test: *versus MSCs + MNCs + PMA + ionomycin; **P <0.05 for the groups indicated in the figure. CM, central memory; EM, effector memory.

**Figure 3:**
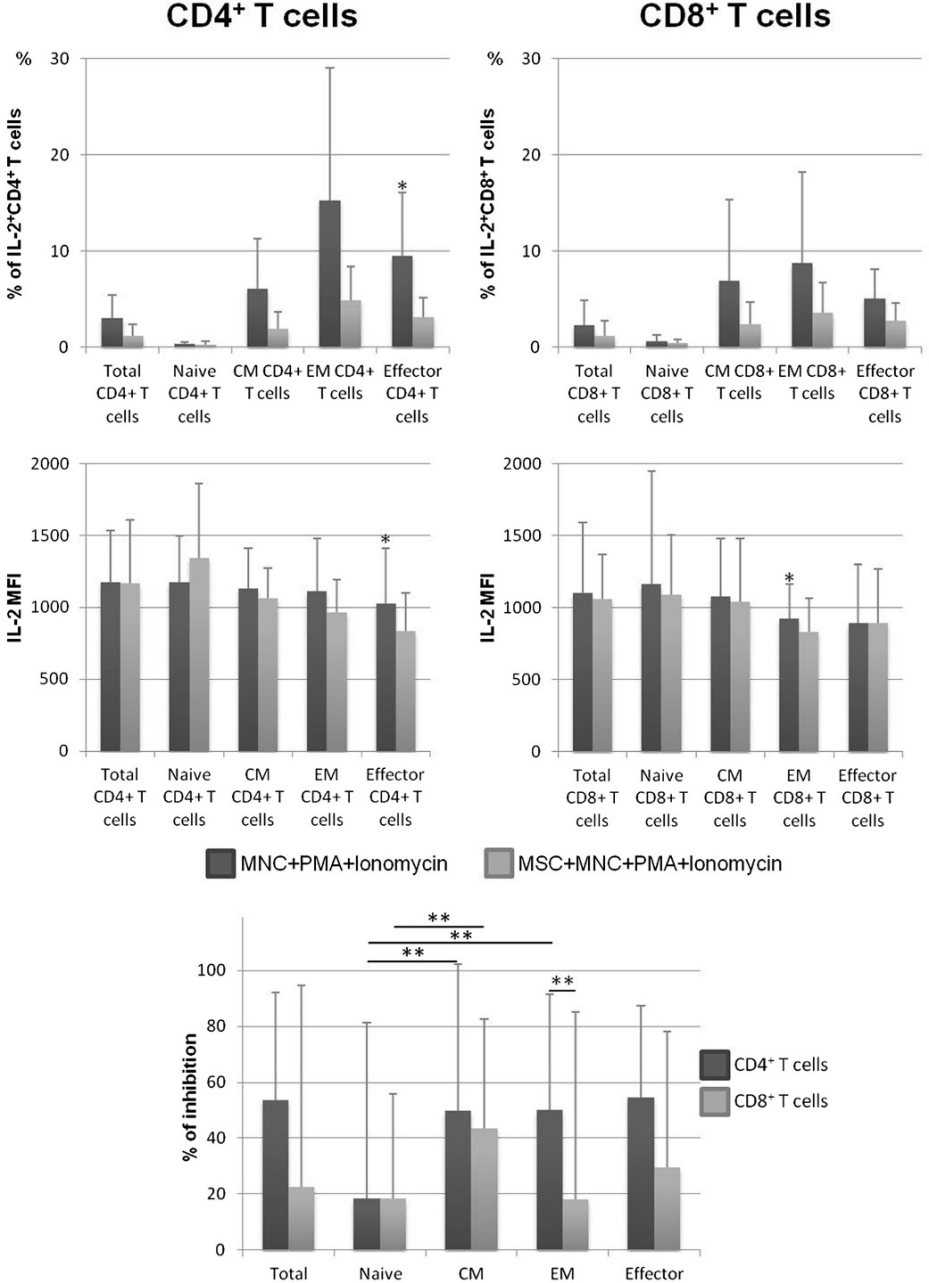
**Percentage of CD4** ^**+**^ **and CD8** ^**+**^ **T cells expressing interleukin-2 (IL-2).** The upper panels show the percentage (mean ± standard deviation) of CD4^+^ and CD8^+^ T cells, distributed among their functional compartments, producing IL-2 (within the correspondent functional compartment of CD4^+^ or CD8^+^ T cells, respectively) and amount of protein expressed per cell, measured as mean fluorescence intensity (MFI) (mean ± standard deviation), after mononuclear cell (MNC) stimulation with phorbol myristate acetate (PMA) plus ionomycin in the absence of mesenchymal stromal cells (MSCs) (MNCs + PMA + ionomycin) or in co-culture with MSCs (MSCs + MNCs + PMA + ionomycin). The lower panel represents the percentage of inhibition induced by MSCs on the percentage of CD4^+^ and CD8^+^ T cells expressing IL-2. Statistically significant differences were considered when *P* <0.05 for Wilcoxon paired-sample test: *versus MSCs + MNCs + PMA + ionomycin; **P <0.05 for the groups indicated in the figure. CM, central memory; EM, effector memory.

Concerning IFNγ and IL-2, we observed an overall reduction of the frequency of both CD4^+^ and CD8^+^ T cells producing these cytokines in MSCs + MNCs + PMA + ionomycin co-cultures (*P* <0.05 for all functional compartments of CD8^+^ T cells producing IFNγ and for effector CD4^+^ T cells producing IL-2), as illustrated in Figures 2 and 3. Of note, MSCs decreased the percentage of IFNγ-producing CD4^+^ and CD8^+^ T cells in 63% and 88% of the individuals under study, respectively, and in 75% and 63% of the individuals for CD4^+^ and CD8^+^ T cells expressing IL-2, respectively. This decrease affected all T-cell compartments but was less pronounced in naive T cells and not visible for naive CD4^+^ T cells producing IFNγ (Figures 2 and 3). MSCs diminished the amount of IFNγ produced by T cells (measured as MFI), primarily in EM and effector compartments (*P* <0.05 for EM CD4^+^ T cells, EM CD8^+^ T cells, and effector CD8^+^ T cells); the effect was more pronounced among CD8^+^ than CD4^+^ T cells (Figure 2). In turn, concerning the amount of IL-2 produced, effector CD4^+^ T cells from MSCs + MNCs + PMA + ionomycin co-cultures displayed a lower IL-2 MFI compared with those from stimulated MNC cultures (*P* <0.05), whereas no differences were found in the remaining T-cell compartments (Figure 3).

We observed no cytokine production by CD4^+^ nor CD8^+^ T cells in non-stimulated MNCs and non-stimulated MNCs + MSCs cultures.

### Mesenchymal stromal cells decrease the frequency of T cells producing IL-17, IL-6, and IL-9

**Figure 4:**
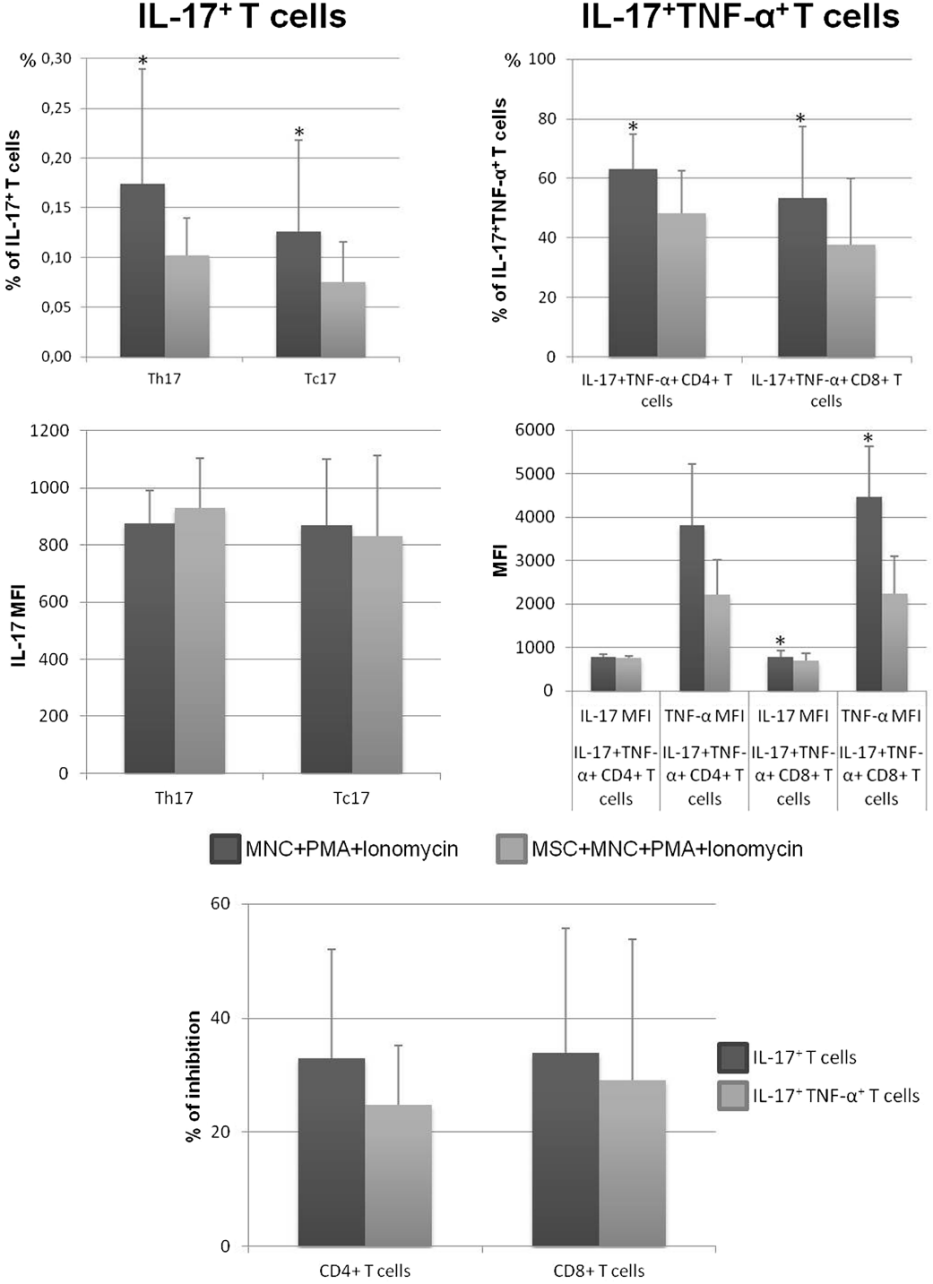
**Percentage of CD4** ^**+**^ **and CD8** ^**+**^ **T cells expressing interleukin-17 (IL-17).** The left upper panels show the percentage (mean ± standard deviation) of CD4^+^ and CD8^+^ T cells producing IL-17 (within total CD4^+^ T cells and total CD8^+^ T cells, respectively) and amount of protein expressed per cell, measured as mean fluorescence intensity (MFI) (mean ± standard deviation), after mononuclear cell (MNC) stimulation with phorbol myristate acetate (PMA) plus ionomycin in the absence of mesenchymal stromal cells (MSCs) (MNCs + PMA + ionomycin) or in co-culture with MSCs (MSCs + MNCs + PMA + ionomycin). The right upper panels show the percentage (mean ± standard deviation) of CD4^+^ and CD8^+^ T cells producing simultaneously IL-17 and tumor necrosis factor-alpha (TNF-α) (within IL-17^+^CD4^+^ T cells and IL-17^+^CD8^+^ T cells, respectively) and amount of protein expressed per cell, measured as MFI (MFI, mean ± standard deviation), after MNC stimulation with PMA plus ionomycin in the absence of MSCs (MNCs + PMA + ionomycin) or in co-culture with MSCs (MSCs + MNCs + PMA + ionomycin). The lower panel represents the percentage of inhibition induced by MSCs on the percentage of CD4^+^ and CD8^+^ T cells expressing IL-17 or IL-17 and TNF-α. Statistically significant differences were considered when *P* <0.05 for Wilcoxon paired-sample test: *versus MSCs + MNCs + PMA + ionomycin. CM, central memory; EM, effector memory.

The presence of MSCs in the stimulated-MNC culture resulted in a decreased percentage of both Th17 and Tc17 cells (*P* <0.05), verified in all individuals studied, without alteration of the amount of IL-17 produced per cell, measured as MFI (Figure 4). Similarly, MSCs reduced the frequency IL-17^+^TNF-α^+^CD4^+^ and IL-17^+^TNF-α^+^CD8^+^ T cells (*P* <0.05), which was observed in all individuals under study. In these T-cell populations, IL-17 MFI remained constant, whereas a lower TNF-α MFI was detected in both cell populations for MSCs + MNCs + PMA + ionomycin co-cultures (*P* <0.05 for CD8^+^ T cells; Figure 4).

**Figure 5:**
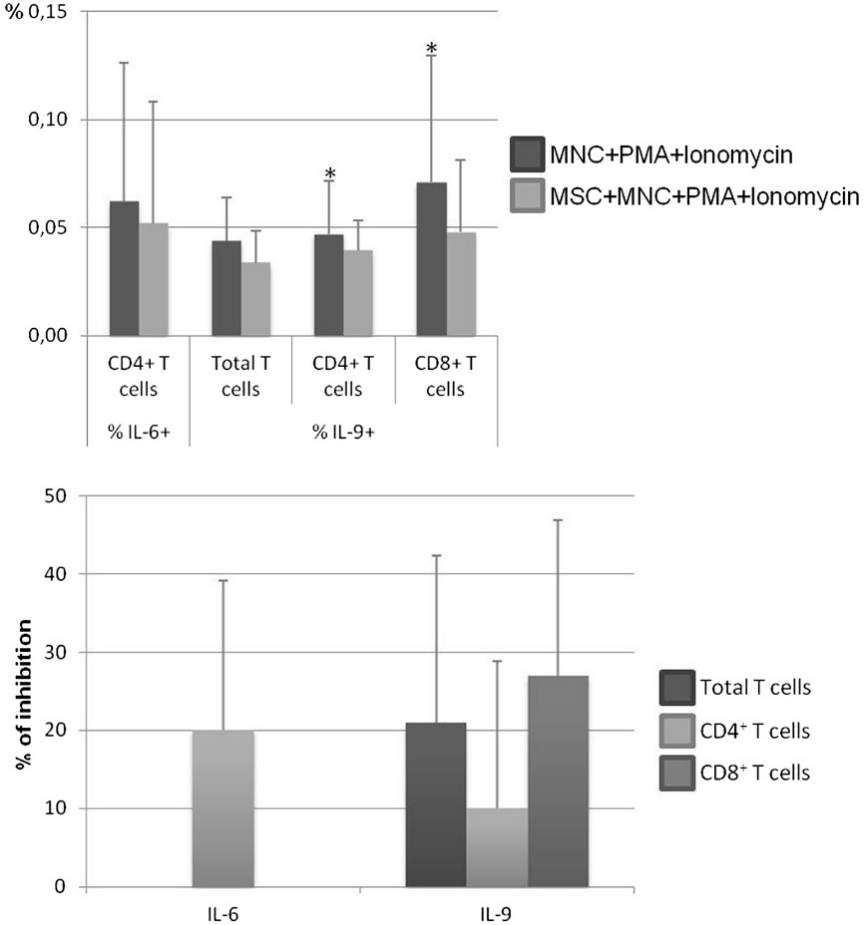
**Percentage of CD4** ^**+**^ **and CD8** ^**+**^ **T cells expressing interleukin-6 (IL-6) or IL-9.** The upper panel shows the percentage (mean ± standard deviation) of CD4^+^ T cells expressing IL-6 (within total CD4^+^ T cells) and of total, CD4^+^, and CD8^+^ T cells producing IL-9 (within total T cells, CD4^+^ T cells, and total CD8^+^ T cells, respectively), after mononuclear cell (MNC) stimulation with phorbol myristate acetate (PMA) plus ionomycin in the absence of mesenchymal stromal cells (MSCs) (MNCs + PMA + ionomycin) or in co-culture with MSCs (MSCs + MNCs + PMA + ionomycin). The lower panel shows the percentage of inhibition induced by MSCs on the percentage of total, CD4^+^ and/or CD8^+^ T cells expressing IL-6 or IL-9. Statistically significant differences were considered when *P* <0.05 for Wilcoxon paired-sample test: *versus MSCs + MNCs + PMA + ionomycin.

The frequency of CD4^+^ and CD8^+^ T cells expressing IL-6 or IL-9 was lower in the presence of MSCs (*P* <0.05 for IL-9^+^CD4^+^ T cells and IL-9^+^ CD8^+^ T cells); 88% of individuals showed a decreased percentage of CD4^+^ T cells expressing IL-6, while in 75% and 100% of the individuals, MSCs reduced CD4^+^ and CD8^+^ T cells producing IL-9, respectively (Figure 5).

We observed no cytokine production by CD4^+^ or CD8^+^ T cells in non-stimulated MNCs and non-stimulated MNCs + MSCs cultures.

### mRNA expression of IL-4, IL-10, TGF-β, and CTLA4 in purified CD4^+^ and CD8^+^ T cells from MNCs cultured in the absence/presence of mesenchymal stromal cells

**Figure 6:**
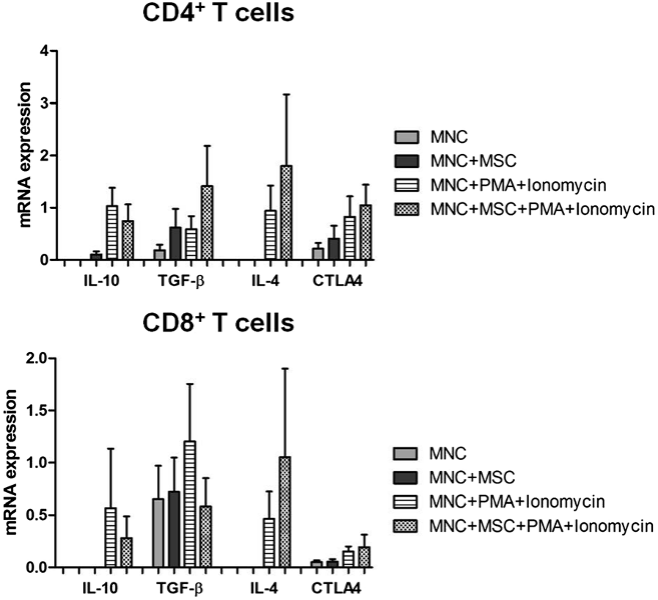
**mRNA expression of interleukin-10 (IL-10), transforming growth factor-beta (TGF-β), IL-2, IL-4, and CTLA-4 in CD4** ^**+**^ **and CD8** ^**+**^ **T cells.** The figure shows the results of a semi-quantitative analysis of IL-10, TGF-β, IL-2, IL-4, and CTLA-4 (cytotoxic T-lymphocyte-associated protein 4) mRNA expression in fluorescence-activated cell sorting (FACS)-purified CD4^+^ and CD8^+^ T cells from the following culture conditions: non-stimulated mononuclear cells (MNCs), non-stimulated MNCs co-cultured with mesenchymal stromal cells (MSCs) (MNCs + MSCs), MNCs stimulated with phorbol myristate acetate (PMA) plus ionomycin (MNCs + PMA + ionomycin), and MNCs stimulated with PMA plus ionomycin in co-culture with MSCs (MNCs + MSCs + PMA + ionomycin). Statistically significant differences were considered when *P* <0.05 for Wilcoxon paired-sample test, comparing MNCs + PMA + ionomycin versus MNCs + MSCs + PMA + ionomycin.

Overall, MSCs decreased IL-10 and increased IL-4 mRNA expression in stimulated CD4^+^ and CD8^+^ T cells, while TGF-β mRNA levels were reduced in CD8^+^ and augmented in CD4^+^ T cells, and CTLA4 remained constant regardless of MSCs presence in the MNCs culture (Figure 6). Of note, a high inter-individual variability in mRNA levels of all molecules studied was observed.

### Protein and mRNA expression in phorbol myristate acetate plus ionomycin stimulated versus non-stimulated mesenchymal stromal cells

**Figure 7:**
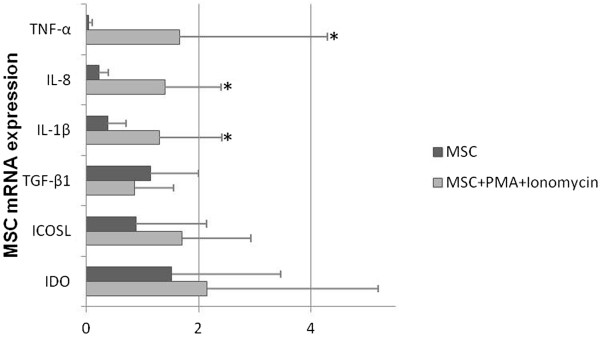
**mRNA expression of TNF-α, IL-8, IL-1β, TGF-β, ICOSL, and IDO in MSCs.** Semi-quantitative analysis of TNF-α, IL-8, IL-1β, TGF-β, ICOSL, IDO mRNA expression in non-stimulated MSCs (MSCs) and in MSCs stimulated with PMA plus ionomycin (MSCs + PMA + ionomycin). Statistically significant differences were considered when *P* <0.05 for Wilcoxon paired-sample test, comparing MSCs versus MSCs + PMA + ionomycin. ICOSL, inducible costimulatory ligand; IDO, indoleamine-2,3-dioxygenase; IL, interleukin; MSC, mesenchymal stromal cell; PMA, phorbol myristate acetate; TGF-β, transforming growth factor-beta; TNF-α, tumor necrosis factor-alpha.

The analysis of the protein expression of CD13, CD44, CD73, CD90, CD106, and CD184 (CXCR4) by flow cytometry showed no differences with biological significance for these molecules (data not shown), although there were significant statistical differences for CD106 (MFI of 2,499 ± 861 and 2,713 ± 869 for non-stimulated and stimulated MSCs, respectively) and CD184 (MFI of 7,103 ± 2,059 and 8,116 ± 2,224 for non-stimulated and stimulated MSCs, respectively). In turn, MSCs stimulation with PMA plus ionomycin induced the increase of IDO, ICOSL, IL-1β, IL-8, and TNF-α mRNA levels (*P* <0.05 for IL-1β, IL-8, and TNF-α) and showed a tendency to decrease TGF-β mRNA (not statistically significant), as illustrated in Figure 7.

## Discussion

Despite the increasing number of studies on MSC-derived immunosuppression of T cells, those which investigated whether MSCs action differed among T-cell functional compartments are scarce. As the immune system disorder candidates to MSCs cell therapy comprise a heterogeneous group concerning the distribution of T cells among their functional compartments and the effector T cells underlying the disease, it becomes urgent to understand how MSCs regulate the distinct T-cell functional compartments.

Thus, in the present study, the influence of MSCs in the cytokine expression profile of naive, CM, EM, and effector compartments of CD4^+^ and CD8^+^ T cells was evaluated directly in these subsets naturally occurring in the peripheral blood of healthy individuals. The abovementioned functional compartments were identified within CD4^+^ and CD8^+^ T-cell populations by flow cytometry on the basis of their differential expression of CD45RA and CD27. The production of IL-2, TNF-α, and IFNγ was further evaluated within each cell compartment. To the best of our knowledge, this is the first study reporting the effect of MSCs on the cytokine expression by T cells that analyzed simultaneously all four functional compartments of CD4^+^ and CD8^+^ T cells naturally occurring in the peripheral blood.

Our results showed that, in a co-culture system with MNCs stimulated with PMA + ionomycin, MSCs downregulate TNF-α, IFNγ, and IL-2 protein expression in both CD4^+^ and CD8^+^ T cells, which corroborates previous studies reporting that MSCs induce the decrease of these cytokines detected in the co-culture medium [22, 25–30] or at the mRNA level [11, 21, 22] or the decrease of the percentage of T cells producing cytokines [14, 20, 29, 31, 32] in *in vitro* experiments and in *in vivo* animal experimental models [18, 19, 33].

The analysis of the expression of those pro-inflammatory cytokines within each T-cell functional compartment revealed that MSCs have the ability to modulate the function of T cells included in naive, CM, EM, and effector compartments but to a different extent. Interestingly, effector CD8^+^ T cells, which present the highest percentage of TNF-α-producing cells among CD4^+^ and CD8^+^ T-cell functional compartments, correspond to those which are less sensitive to MSC-mediated TNF-α downregulation. Therefore, as TNF-α constitutes an important effector mechanism of CD8^+^ T cell-mediated immune response, with a pivotal role in the pathophysiology of several autoimmune disorders and graft-versus-host disease, we may postulate that MSCs present a milder inhibition on cells with a higher degree of differentiation. Conversely, IL-2 expression is highly inhibited in CM, EM, and effector CD4^+^ T cells. MSC-mediated IL-2 downregulation had already been described for CD4^+^ or total T cells and is correlated to MSCs ability to impair T-cell proliferation [11, 21, 28]. Similarly to Aggarwal *et al*. [26], who reported MSC-mediated inhibition of IFNγ in human CD45RA^+^ T cells, we observed a decreased frequency of naive T cells producing IFNγ, but only among CD8^+^ T cells, whereas no inhibitory effect was detected over naive CD4^+^ T cells. Besides, a more pronounced inhibitory effect was observed among the CM, EM, and effector compartments, compared with naive cells, for CD4^+^ and CD8^+^ T cells; similarly, Krampera *et al*. [32] found a stronger inhibitory effect over mouse memory T cells, compared with naive T cells. In the same line, more recent studies showed that MSCs induced human memory T cells, whose identification was based on CD45RO expression, to express IL-17, which did not occur in the naive compartment [21, 23]. At this point, our results and previously published data support the idea that MSCs differently regulate the distinct T-cell functional compartments, which can impact the outcome of MSC therapy.

Concerning other pro-inflammatory cytokines, MSCs induced a decreased percentage of CD4^+^ and CD8^+^ T cells expressing IL-17 and IL-9 and of CD4^+^ T cells producing IL-6. Conversely, at the mRNA level, we observed a tendency to an increase of the anti-inflammatory IL-4 and TGF-β in CD4^+^ T cells, as described by others [1, 18, 26, 29]. The ability to inhibit Th17 differentiation from mouse and human T cells has been attributed to MSCs, and the ability to decrease the expression of IL-17 in differentiated Th17 is an effect assigned to prostaglandin E_2_[14–16, 19, 33, 34]; however, the effect of MSCs on T cells expressing IL-17 is controversial, and some studies showed that Th17 function can be enhanced by MSCs [14, 20–23]. Nevertheless, the different experimental approaches used may explain the conflicting data. Beyond the direct impairment of Th1 and Th17 immune responses, by reducing the expression of their typical effector cytokines (TNF-α, IFNγ, IL-2, and IL-17), MSCs can also induce the expression of the anti-inflammatory cytokine IL-10 in both Th1 and Th17 cells [16, 35].

IL-6 is a cytokine with recognized pro- and anti-inflammatory properties. Roughly, anti-inflammatory biological effects can be ascribed to IL-6 classic signaling via membrane IL-6 receptor (IL-6R), whereas pro-inflammatory function is associated with trans-signaling mediated by soluble IL-6R (sIL-6R) [36]. The essential role of this cytokine in inflammation was confirmed by several animal models of autoimmune inflammatory diseases, where the blockage of IL-6 trans-signaling prevented the inflammatory process. As an inflammatory state contribute to proteolytic cleavage of IL-6R, resulting in the release of sIL-6R, trans-signaling is likely to be enhanced under this condition; besides, IL-6 is necessary for Th17 differentiation and maintenance [37]. MSC-mediated reduction of IL-6 expression in CD4^+^ T cells is in agreement with the decreased expression of the other pro-inflammatory cytokines evaluated in the present study, namely the percentage of T cells producing IL-17.

The biological effects of IL-9 are still poorly described. This cytokine is produced by Th9, Th2, Treg, and Th17 cell subsets and plays an important role in the immune response against helminthes and in allergic inflammation [38–40]. Despite promoting Th2 cytokine production, enhancing the regulatory function of Treg and suppressing the expression of the pro-inflammatory cytokines TNF-α, IL-12, and IFNγ under certain experimental conditions, IL-9 is also able to expand Th17 cell population and to promote inflammation, as the adoptive transfer of Th9-polarized cells induces experimental autoimmune encephalomyelitis and experimental autoimmune uveitis [38, 41]. Recent works showed that *in vivo* systemic administration of MSCs are beneficial in allergic airway inflammation mouse models by increasing IL-10 and decreasing IL-4, IL-5, and IL-13 cytokine secretion [42–45]. To the best of our knowledge, IL-9 had never been evaluated in *in vivo* MSC-mediated suppression of allergic inflammation in animal models; also, this is the first study reporting the inhibition of IL-9-producing CD4^+^ and CD8^+^ T cells by MSCs, opening new possibilities in the investigation of MSCs in allergic inflammation.

## Conclusions

MSCs constitute a possible therapeutic approach for immune-mediated disorders; however, these pathologies are very heterogeneous in what concerns the effector T-cell subset involved and the distribution of T cells among their functional compartments. Thus, a detailed knowledge of the effect of MSCs over each individual functional compartment of CD4^+^ and CD8^+^ T cells became essential to predict and understand their outcome as cell therapy. Here, we investigated whether human BM-MSCs altered the expression of pro-inflammatory cytokines among the CD4^+^ and CD8^+^ T-cell functional compartments naturally occurring in the peripheral blood.

Overall, our results showed that MSCs inhibit TNF-α, IFNγ, and IL-2 protein expression in all CD4^+^ and CD8^+^ T-cell functional compartments, except for IFNγ expression in naive CD4^+^ T cells, where MSCs had no suppressive effect. Interestingly, MSC-derived inhibition was stronger for CD4^+^ than CD8^+^ T cells, concerning IL-2 and TNF-α, but was more pronounced in CD8^+^ T cells for IFNγ.

It is noteworthy that MSCs showed distinct inhibitory patterns for the different functional compartments of T cells. MSC-derived inhibition of TNF-α expression was more marked among naive, CM, and EM T cells for both CD4^+^ and CD8^+^ T-cell subsets; CM CD4^+^ T cells and effector and EM CD8^+^ T cells were the functional compartments displaying a higher degree of suppression of IFNγ expression; and the inhibition of IL-2 production was more pronounced among effector, EM, and CM CD4^+^ T cells and CM CD8^+^ T cells.

Finally, we verified that, under our experimental conditions, MSC co-culture reduced the percentage of both CD4^+^ and CD8^+^ T cells expressing IL-17 and IL-9 and of CD4^+^ T cells producing IL-6. The influence of MSCs on IL-9 can open new possibilities of research regarding the clinical use of MSCs in allergic inflammation.

In the present study, we showed that MSCs differentially regulate the functional compartments of T cells. Also, the pattern of inhibition of the functional compartments of CD4^+^ T cells differed from that observed in CD8^+^ T cells. Our data revealed that the distinctive distribution of T cells among activation/differentiation compartments of immune system disorders will condition the therapeutic effect of MSCs.

## Electronic supplementary material

Additional file 1: Figure S1: Gating strategy for identifying CD4^+^ and CD8^+^ T cells’ functional compartments and cytokine expression among each T-cell compartment. Bivariate dot plot histograms illustrate **(A-C)** overall T cells in the cell culture and (C) CD4^+^ (blue events) and CD8^+^ (pink events) T cells. The identification of T cells’ functional compartments was made within CD8^+^ **(D)** and CD4^+^ **(E)** T-cell subpopulations, based on CD45RA and CD27 expression, as follows: CD45RA^+^CD27^+^ phenotype corresponds to naive (N) T cells, CD45RA^−^CD27^+^ corresponds to central memory (CM), effector memory (EM) T cells are CD45RA^−^CD27^−^, and effector (E) T cells display CD45RA^+^CD27^−^ phenotype. The expression of cytokines—illustrated in **(F)** for CD8^+^ T cells and in **(G)** for CD4^+^ T cells—was evaluated within each CD4^+^ and CD8^+^ T-cell functional compartment. (JPEG 117 KB)

## Abbreviations

BM: bone marrow
CM: central memory
CTLA4: cytotoxic T-lymphocyte-associated protein 4
EM: effector memory
FACS: fluorescence-activated cell sorting
FSC: forward scatter
ICOSL: inducible costimulatory ligand
IDO: indoleamine-2,3-dioxygenase
IFNγ: interferon γ
IL: interleukin
IL-6R: interleukin-6 receptor
mAb: monoclonal antibody
MFI: mean fluorescence intensity
MNC: mononuclear cell
MSC: mesenchymal stromal cell
PBS: phosphate-buffered saline
PCR: polymerase chain reaction
PMA: phorbol myristate acetate
sIL-6R: soluble interleukin-6 receptor
SSC: side scatter
TGF-β: transforming growth factor-beta
TNF-α: tumor necrosis factor-alpha
Treg: regulatory T.
