# Supplementary figures and images for: Effect of human bone marrow mesenchymal stromal cells on cytokine production by peripheral blood naive, memory, and effector T cells
Source: Stem Cell Res Ther. 2015 Jan 5;6(1):3. doi: 10.1186/scrt537 (PMC4417198; doi:10.1186/scrt537)

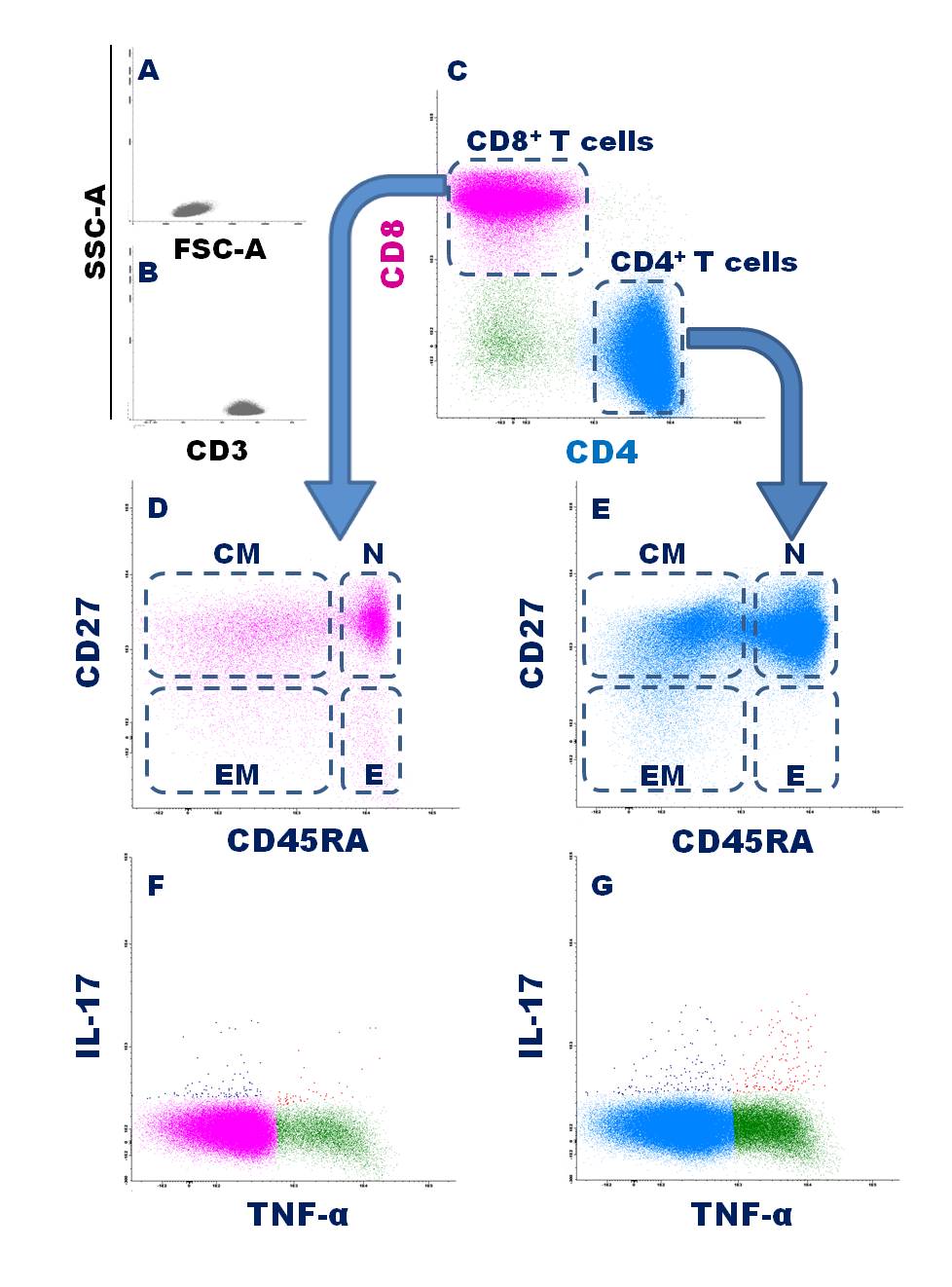

Supplement: Supplementary file 1 — Additional file 1: Figure S1: Gating strategy for identifying CD4+ and CD8+ T cells’ functional compartments and cytokine expression among each T-cell compartment. Bivariate dot plot histograms illustrate (A-C) overall T cells in the cell culture and (C) CD4+ (blue events) and CD8+ (pink events) T cells. The identification of T cells’ functional compartments was made within CD8+ (D) and CD4+ (E) T-cell subpopulations, based on CD45RA and CD27 expression, as follows: CD45RA+CD27+ phenotype corresponds to naive (N) T cells, CD45RA−CD27+ corresponds to central memory (CM), effector memory (EM) T cells are CD45RA−CD27−, and effector (E) T cells display CD45RA+CD27− phenotype. The expression of cytokines—illustrated in (F) for CD8+ T cells and in (G) for CD4+ T cells—was evaluated within each CD4+ and CD8+ T-cell functional compartment. (JPEG 117 KB) [file 13287_2014_419_MOESM1_ESM.jpeg]
